# Streptozocin-Based Chemotherapy in Patients with Advanced Neuroendocrine Neoplasms – Predictive and Prognostic Markers for Treatment Stratification

**DOI:** 10.1371/journal.pone.0143822

**Published:** 2015-12-02

**Authors:** Sebastian Krug, Michael Boch, Hanna Daniel, Wilhelm Nimphius, Daniela Müller, Patrick Michl, Anja Rinke, Thomas Matthias Gress

**Affiliations:** 1 Department of Gastroenterology, University of Marburg, Marburg, Germany; 2 Institute of Medical Biometry, University of Marburg, Marburg, Germany; 3 Institute of Pathology, University of Marburg, Marburg, Germany; 4 Department of Gastroenterology and Hepatology, University of Halle, Halle, Germany; H. Lee Moffitt Cancer Center & Research Institute, UNITED STATES

## Abstract

**Background and Aim:**

Chemotherapy with streptozocin (STZ) in combination with 5-FU or doxorubicin (Dox) represents a standard of care for patients with metastatic pancreatic neuroendocrine neoplasms (pNEN). However, predictive markers for patient selection are still missing. The aim of this study was a retrospective evaluation of the clinicopathological characteristics of pNEN patients receiving STZ-based chemotherapies and to identify predictive and prognostic markers.

**Patients and Methods:**

We retrospectively analyzed 77 patients treated at our center between 1995 and 2013. The median overall survival (OS) and progression-free survival (PFS) were calculated using Kaplan-Meier and Cox regression methods, respectively. Uni- and multivariate analyses were performed.

**Results:**

The median PFS (mPFS) in patients receiving STZ/5-FU/Dox was 16 months with a median OS (mOS) of 28 months. Objective response rate (ORR) and disease control rate (DCR) were 34% and 72%, respectively. Biochemical response and positive octreotide scintigraphy predicted objective response. Univariate analysis revealed Ki-67 > 10% and the absence of biochemical or objective response by imaging as independent risk factors for shorter PFS. Additionally, performance status (PS) and resection of the primary tumor were observed to influence mOS. Treatment was well tolerated with less than 10% grade 3 and 4 toxicities.

**Conclusions:**

STZ-based chemotherapy is an effective and well-tolerated treatment option in patients with well differentiated neuroendocrine neoplasms. Positive octreotide scintigraphy and biochemical response predict objective response.

## Introduction

Neuroendocrine tumors (NETs) are a heterogeneous group of neoplasms with increasing incidence [1] originating from endocrine cells in different anatomic locations. Pancreatic NETs differ from intestinal NETs in many aspects including clinical presentation with distinct hormone syndromes, genetic findings (e.g. mutations in the Menin gene [2]), a more aggressive course of disease resulting in worse prognosis [1], and responsiveness to treatment modalities such as molecular targeted agents and chemotherapy.

While the results of chemotherapy in patients with intestinal NETs are disappointing resulting in objective response rates of less than 20% in most trials, pancreatic NETs were shown to be chemosensitive. The combination of streptozocin (STZ) and fluorouracil (5-FU) is recommended as standard treatment for metastatic pancreatic NETs in European guidelines [3, 4].

STZ is available since the early 80ies and approved for the treatment of pancreatic NETs in several countries. The early prospective randomized trials by Moertel reported high response rates (RR) of STZ-based combinations exceeding 60% [5, 6]. However, two subsequent retrospective series failed to confirm these results, which was attributed the definition of response [7, 8]. More recently, larger retrospective studies using standardized radiological response criteria repeatedly reported RR´s ranging between 30 and 40% for STZ-based combination treatments [9–11].

A variety of prognostic factors has been described for patients with NETs including age, performance status, stage according to ENETS [12, 13] and AJCC [14], tumor load, levels of chromogranin A (CgA) [15], presence of circulating tumor cells [16] and grading based on the proliferation marker Ki-67.

The latest WHO classification [17] of NETs is based on Ki-67 values and the prognostic relevance of this grading system has been validated in several studies [12, 18–20]. In contrast, the predictive value of Ki-67 is less clear. To date, no established predictive markers are available to facilitate treatment decisions. The ESMO guideline recommends the use of STZ-based chemotherapy in patients with pancreatic NETs and a proliferation rate between 5 and 20% [4]. However, this represents an expert opinion which is not evidence-based, since most chemotherapy trials in pNEN published so far did not assess the role of Ki-67 as predictive marker. Only in one study by O´Toole and co-workers an association between Ki-67 levels >5% and lack of response to systemic chemotherapy was reported [21]. It was thus the aim of our study to identify prognostic and predictive markers for pNEN-patients treated with STZ-based chemotherapy at our center.

## Patients and Methods

### Patients

77 consecutive patients with histologically confirmed pancreatic neuroendocrine tumors who received STZ-based chemotherapy between 1995 and 2013 were retrospectively identified from a database at the comprehensive cancer center at the University Hospital of Marburg.

This study was conducted in accordance with the Declaration of Helsinki. Collection, storage, and evaluation of patient-related information in our NEN database were performed with the approval of the local ethics committee at the University of Marburg and after obtaining the patients informed consent. The initial statement of the local ethics committee was that a formal approval and written informed consent for collection and analysis of data arising from the routine clinical evaluation within the own hospital was not required. Therefore, patients who had their last visit/ died before 2004 only were asked for verbal consent (with approval of the ethics committee of this consent procedure). In 2004 the German NET registry was built up and for transmission of pseudonymized data a written approval of the ethics committee was obtained. Since then all patients additionally gave a written consent for data collection and analysis.

### Protocol treatment and toxicity assessment

All patients received STZ-containing chemotherapy in combination with Doxorubicin (Dox) or 5-FU. For patients who initially received chemotherapy with STZ/Dox, Dox was replaced by 5-FU before the cumulative cardiotoxic dose of 550mg/m^2^ Dox was reached. The chemotherapeutic STZ/Dox regimen included STZ at a dose of 500 mg/m^2^ on day 1–5 and Dox at a dose of 50 mg/m² on day 1 and 22. The regimen was repeated every 6 weeks. The STZ/5-FU regimen included short-term infusion of 5-FU at a dose of 400 mg/m^2^ on day 1–5, in addition to STZ at a dose of 500 mg/m^2^ on day 1–5 every 6 weeks. In case of impaired performance status or toxicity a delay of 2 weeks was allowed. Concomitant treatment with somatostatin analogs were allowed for patients suffering from significant hormone excess syndromes.

Adverse effects were recorded during each cycle of chemotherapy, and reported using CTCAE version 3.0 (http://ctep.cancer.gov).

### Follow up and evaluation of tumor response

Follow-up investigations were scheduled after 3 completed treatment courses including history, physical examination, laboratory investigations and imaging (CT or MRI scan). Biochemical evaluation included chromogranin A (CgA) measurements. In addition, the surveillance schedule included a somatostatin receptor scintigraphy every 18–24 months in the majority of cases.

Response to treatment was evaluated in this study using the international criteria proposed by the Response Evaluation Criteria in Solid Tumors (RECIST) Committee [22]. The biochemical response was defined as a reduction of more than 30% compared to baseline levels.

### Immunohistochemistry

The Ki-67 proliferation index was evaluated using a standardized protocol with the Leica-Bond-Max-Autostainer and the Dako antibody in a 1:1000 dilution. Classification was done according to the revised WHO classification of tumors of the digestive system 2010 [23]. Ki-67 values found in the pathologic reports and in some cases when the Ki-67 value was not available in the reports, from new Ki-67 stainings of archival tissue specimens were used. Tumors were graded using the proposed grading system of Rindi et al [23, 24]. The analyses were performed by one pathologist with expertise in endocrine and pancreatic tumors (W.N.) in a blinded fashion regarding tumor parameters.

### Statistical design and analysis

The comparisons between response and tumor characteristics, disease extension, or laboratory features were based on Chi-square and Fisher’s exact tests, as appropriate. PFS was measured from the beginning of treatment to progression, death, or last follow-up. OS was measured from the beginning of treatment to the time of last follow-up or death. Actuarial survival was measured by the method of Kaplan and Meier [25]. The statistical differences in PFS between groups of patients were estimated by the log-rank test [26]. The statistical independence between prognostic variables was evaluated by multivariate analysis using the Cox proportional hazard model [27]. All statistical calculations were performed using SPSS (IBM SPSS Statistics). Differences were considered statistically significant when the P value was less than 0.05.

## Results

### Patient characteristics

We identified 77 patients who received STZ in the time period between 1995 and 2013. Baseline characteristics are listed in Table 1. The majority of patients had pancreatic neuroendocrine neoplasms (n = 65, 84.4%), while 12 (15.6%) patients had NEN from non-pancreatic origin, among them 8 bronchial NEN (10.4%), 3 CUPs (3.9%) and 1 duodenal (1.3%) NEN. There were 55 (71.5%) non-functioning and 21 (27.3%) functioning tumors. Information about tumor differentiation and grading were available in 70 (90.9%) and 67 (87%) patients, respectively. Among these, 68 tumors (88.3%) were well differentiated including 9 (11.7%) G1 and 51 (66.2%) G2 tumors, and 5 well differentiated tumors albeit with Ki-67 proliferation rates >20% (so-called NET G3). For 3 older samples of well differentiated tumors grading and Ki-67 were not documented and could not be determined since no tissue was available anymore. Finally, two neuroendocrine carcinomas (2.6%) were documented. 88.3% patients had liver (n = 68) and 66.2% lymph node (n = 51) metastases. Bones represented the third most common site of metastases (n = 30, 39%). Somatostatin receptor status determined by octreotide scintigraphy was positive in 56 patients (72.7%) and negative in 14 patients.

**Table 1:** Clinicopathological features of patients (N = 77).

Characteristics	No. of patients	%
**Gender**		
**male**	39	50.6
**female**	38	49.4
**Age at diagnosis in years**		
**median**	53	
**range**	25–79	
**WHO PS**		
**0**	38	49.4
**1**	32	41.6
**unknown**	7	9.1
**Primary tumor location**		
**Pancreas**	65	84.4
**Non-Pancreas**	12	15.6
**Bronchus**	8	10.4
**CUP**	3	3.9
**Duodenum**	1	1.3
**Tumor type**		
**Functioning**	21	27.3
**Insulinoma**	7	9.1
**Gastrinoma**	6	7.8
**Glucagonoma**	2	2.6
**VIPoma**	4	5.2
**ACTHoma**	2	2.6
**Nonfunctioning**	55	71.5
**unknown**	1	1.3
**Differentiation**		
**NET**	68	88.3
**NEC**	2	2.6
**unknown**	7	9.1
**Grading**		
**G1**	9	11.7
**G2**	51	66.2
**G3**	7	9.1
**unknown**	10	13.0
**Sites of metastases**		
**Lymph Node**	51	66.2
**Liver**	68	88.3
**Bone**	30	39.0
**Others**	31	40.3
**Octreotide scintigraphy**		
**positive**	56	72.7
**negative**	14	18.2
**unknown**	7	9.1

Abbreviations: PS = performance status, CUP = carcinoma of unknown primary, G = grading, VIP = vasoactive intestinal polypeptide, ACTH = adrenocorticotropic hormone, NET = neuroendocrine tumor, NEC = neuroendocrine carcinoma.

Treatment details are listed in Table 2. 23 patients (29.9%) underwent primary tumor resection and 14 patients (18.2%) received synchronous resection of liver metastases. Only 15 patients (19.5%) were treatment-naive when chemotherapy was started. Most patients had one (n = 32, 41.6%) or two (n = 30, 39%) prior systemic or liver directed treatments. Therefore, median time to STZ-chemotherapy was 33 months (range 1–181). In our cohort, 31 patients received STZ and Dox (40.3%), 30 patients received STZ and 5-FU (39%) and 13 patients received STZ and 5-FU (16.9%) after STZ and Dox in a sequential approach. The median number of cycles administered was 3 (range 1–6) and 4 (range 1–12) for STZ/ Dox and STZ/ 5-FU, respectively.

**Table 2:** Clinicopathological features of patients (N = 77).

**Characteristics**	**No. of patients**	**%**
**Primary tumor resection**		
** yes**	23	29.9
** no**	48	62.3
** unknown**	6	18.2
** synchronic liver metastases resection**	14	7.8
**Age at CTx in years**		
** Median**	56	
** Range**	27–77	
**Time to CTx in months**		
** Median**	33	
** Range**	1–181	
**Chemotherapy No.**	**No. of patients**	
** STZ/Dox**	31	40.3
** STZ/5-FU**	30	39
** sequential approach**	13	16.9
**Median No. of cycles administered**		
**STZ/Dox**		
** Median**	3	
** Range**	1–6	
**STZ/5-FU**		
** Median**	4	
** Range**	4–12	
**No. prior systemic / liver directed therapies**	**No. of patients**	
** 0**	15	19.5
** 1**	32	41.6
** ≥2**	30	39.0

Abbreviations: CTx = chemotherapy, Dox = doxorubicin, STZ = streptozocin.

### Safety

Sixty-six patients were assessable for toxicities (Table 3). Altogether, 228 events were documented during and after chemotherapy including 20 grade 3 and 4 adverse events (8.8%). In brief, most common adverse events comprised grade 1 or 2 hematological (n = 67, 29%), hepatological (n = 40, 17.5%), gastrointestinal (n = 33, 14.5%) and renal (n = 31, 13.6%) toxicity. Less than 10% grade 3 or 4 toxicities occurred including leukopenia (n = 3, 1.3%) and nausea or vomiting (n = 5, 2.2%). A total number of 20 events (8.8%) occurred due to hair loss mostly following Dox treatment. In contrast, renal adverse events including proteinuria and/or renal failure were typical side effects of STZ infusion. Other adverse events (n = 22, 9.6%) included infectious, neurological and cardiac toxicities of which 5 were grade 3 cardiac toxicities (2.2%).

**Table 3:** Toxicities of chemotherapy (in 66 evaluable patients).

Toxic reaction	Grade 1	%	Grade 2	%	Grade 3	%	Grade 4	%
**Hematologic**								
**Leukopenia**	20	30.3	18	27.3	3	4.5	0	0
**Thrombocytopenia**	6	9.1	2	3.0	0	0	0	0
**Anemia**	15	22.7	6	9.1	0	0	0	0
**Gastrointestinal**								
**Nausea or Vomiting**	12	18.2	8	12.1	5	7.6	1	1.5
**Diarrhea/Obstipation**	7	10.6	6	9.1	0	0	0	0
**Mucositis**	0	0	0	0	1	1.5	0	0
**Hepatologic**	31	47.0	9	13.6	2	3.0	1	1.5
**Renal**	27	40.9	4	6.1	2	3.0	0	0
**Hair loss**	4	6.1	16	24.2	0		0	0
**Other**	10	15.2	7	10.6	5	7.6	0	0

### Therapy efficacy, predictive and prognostic indicators for PFS and OS

**Fig 1:**
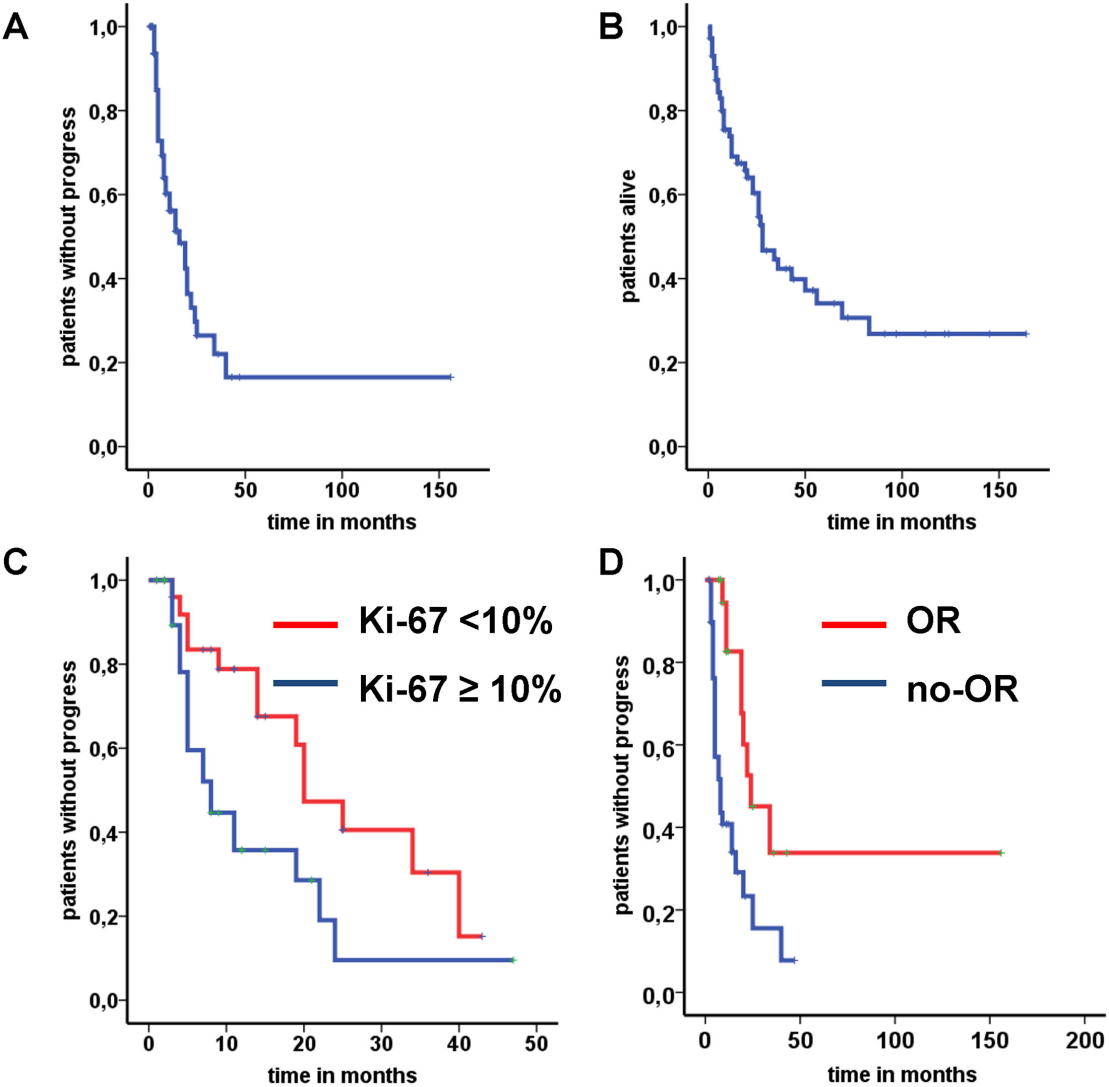
Kaplan Meier survival analyses in patients treated with streptozocin. (A) Progression-free survival (mPFS = 16 months) for the entire group of patients (n = 64). (B) Overall survival (mOS = 28 months) for the entire group of patients (N = 64). (C) Association between Ki-67 and mPFS (cut-off 10%; 20 vs. 8 months, P = 0.015; N = 60) (D) Association between objective response (OR) and mPFS (20 vs 4 months, P < 0.001; N = 62).

For 64 patients (83.1%) the radiologic response was assessable (Table 4). 22 patients (34.4%) experienced an objective response including 2 (3.1%) complete (CR) and 20 (31.3%) partial responses (PR). Disease control was achieved for a high proportion (71.9%) of patients comprising patients with response (PR and CR) and with stable disease (n = 24, 37.5%). Progressive disease was documented in 18 patients (28.1%). Median PFS and median OS were calculated as 16 and 28 months, respectively (Fig 1).

**Table 4:** Response and predictive markers (in 64 evaluable patients).

Predictor	CR	PR	SD	PD	ORR %	DCR %	Fisher’s exact test		X2-test for trend	
Number of patients	2	20	24	18	34.4	71.9	OR	DC	OR	DC
**Primary tumor location**							P value		P value	
**Pancreas**	2	17	23	14	33.9	75.0	1	0.095	0.89	0.076
**Non-Pancreas**		2	1	4	28.6	42.9				
**Site of metastases**										
**Liver+Lymphnode**	1	10	10	6	40.7	77.8	0.26	0.4	0.21	0.3
**Liver+LN+other**		9	14	12	25.7	65.7				
**Ki-67 in %**										
**<2**		3	5	1	33.3	88.9	G1 vs G2/3		0.28	0.058
**2–20**	2	16	17	13	37.5	72.9	1	0.42	0.85	0.22
**>20**		1		3	25.0	25.0				
**Age before CTx**										
**<60**	1	6	12	6	28.0	76.0	0.59	0.77	0.42	0.62
**>60**	1	13	12	11	37.8	70.3				
**PS**										
**0**	2	13	12	10	40.5	73.0	0.105	0.78	0.089	0.67
**≥1**		5	12	8	20.0	68.0				
**Octreotide scintigraphy**										
**positive**	2	18	18	12	40.0	76.0	0.046	0.28	0.037	0.22
**negative**		1	6	5	8.3	58.3				
**Biochemical response**										
**yes**	1	13	5	10	48.3	65.5	0.052	0.082	0.027	0.066
**no**	1	5	18	5	20.7	82.8				
**Chromogranin A**										
**>50 U/l**	1	15	15	14	35.6	68.9	1	0.42	0.9	0.22
**≤50 U/l**	1	2	5	1	33.3	88.9				
**Primary tumor operation**										
**yes**	1	7	5	8	38.1	61.9	0.58	0.25	0.57	0.24
**no**	1	12	19	10	31.0	76.2				

Abbreviations: CR = complete response, PR = partial response, SD = stable disease, PD = progressive disease, ORR = objective response rate, DCR = disease control rate, LN = lymph node, others = bone, lung, cerebral, peritoneal, lienal and adrenal gland, CTx = chemotherapy, PS = performance status.

A panel of clinicopathological parameters was evaluated for their association to objective response (OR) as putative predictive markers (Table 4). Out of these, only a positive SMS receptor status, (positive vs. negative: 40.0% vs. 8.3%, P = 0.037) as well as biochemical response (positive vs. negative: 48.3% vs. 20.7%, P = 0.027) correlated with objective response as determined by imaging.

Univariate analyses were performed evaluating Ki-67, performance status (PS), primary tumor resection, objective response (OR), disease control (DC), biochemical response and prior therapies to identify the correlation to PFS and OS (Table 5). Statistically significant risk factors for disease progression included Ki-67 ≥10% (HR 2.3, 95% CI 1.1–4.8, P = 0.022), whereas OR (HR 0.3, 95% 0.2–0.7, P = 0.002) and presence of a biochemical response (HR 0.4, 95% CI 0.2–0.9, P = 0.035) significantly decreased the risk for disease progression. There was no statistical association between PFS and performance status, primary tumor resection or prior therapies.

**Table 5:** Univariate analysis for prognostic indicators.

	PFS			OS		
Variable	HR	95% CI	P-value	HR	95% CI	P-value
**Ki-67**						
**<10%**	1		**0.022**	1		**0.024**
**≥10%**	2.3	1.1–4.8		2.3	1.1–4.7	
**PS**						
**0**	1		0.17	1		**< 0.001**
**≥1**	1.6	0.8–3.1		3.5	1.8–6.9	
**Primary tumor resection**						
**no**	1		0.72	1		**0.034**
**yes**	1.1	0.6–2.2		0.5	0.2–0.9	
**OR**						
**no**	1		**0.002**	1		0.11
**yes**	0.3	0.2–0.7		0.5	0.3–1.1	
**DC**						
**no**				1		**0.01**
**yes**				0.4	0.2–0.8	
**Biochemical response**						
**no**	1		**0.035**	1		0.13
**yes**	0.4	0.2–0.9		0.5	0.2–1.2	
**Prior therapies**						
**≤1**	1		0.12	1		0.72
**>1**	0.5	0.3–1.2		0.9	0.4–1.8	

Abbreviations: PS = performance status, OR = objective response, DC = disease control, HR = Hazard ratio, mPFS = median progression-free survival, mOS = median overall survival, CI = confidence interval.

Prognostic indicators for reduced OS were Ki-67 ≥10% (HR 2.3, 95% CI 1.1–4.7, P = 0.024) and PS ≥1 (HR 3.5, 95% CI 1.8–6.9, P = < 0.001). In contrast, primary tumor resection (HR 0.5, 95% CI 0.2–0.9, P = 0.034) and radiological disease control (HR 0.4, 95% CI 0.2–0.8, P = 0.01) were identified as relevant favourable prognostic indicators.

Interestingly, there was no correlation between OS and OR or biochemical response. In addition, multivariate analysis revealed that only a Ki-67-value ≥10% (HR 2.9, 95% CI 1.1–7.8, P = 0.034) was an independent prognostic marker for PFS (Table 6). However, no association between the risk factors used for univariate analyses and OS was found.

**Table 6:** Multivariate analysis for prognostic indicators.

	PFS			OS		
Variable	HR	95% CI	P-value	HR	95% CI	P-value
**Ki-67**						
**<10%**	1		**0.034**	1		0.11
**≥10%**	2.9	1.1–7.8		2.3	0.8–6.2	
**PS**						
**0**	1		0.72	1		0.076
**≥1**	1.2	0.5–2.6		2.6	0.9–7.3	
**Primary tumor resection**						
**no**	1		0.24	1		0.15
**yes**	0.6	0.2–1.4		0.4	0.1–1.4	
**OR**						
**no**	1		0.08	1		0.65
**yes**	0.4	0.1–1.1		0.8	0.2–2.5	
**DC**						
**no**				1		0.2
**yes**				0.5	0.1–1.5	
**Biochemical response**						
**no**	1		0.69	1		0.22
**yes**	1.3	0.4–4.4		0.5	0.1–1.6	
**Prior therapies**						
**≤1**	1		0.5	1		0.9
**>1**	0.7	0.3–1.9		1	0.3–3.1	

Abbreviations: PS = performance status, OR = objective response, DC = disease control, HR = Hazard ratio, PFS = progression-free survival, OS = overall survival, CI = confidence interval.

Representative Kaplan-Meier curves for Ki-67 (mPFS 20 vs. 8 months, P = 0.015) and OR (mPFS 24 vs. 8 months, P = 0.001) are shown in Fig 1C and 1D to visualize the impact of these parameters on PFS.

## Discussion

In this study, we confirmed that STZ-based regimens in combination with Dox or 5-FU are safe and effective treatment options in a large, retrospective cohort of patients mostly with advanced pancreatic neuroendocrine neoplasms. Consistent with prior studies, chemotherapy induced an ORR and DCR of 34% and 72%, respectively, translating into a mPFS of 16 months and mOS of 28 months. Beyond well-characterized prognostic indicators including Ki-67 and biochemical response for PFS, we identified biochemical response and positive SMS scintigraphy as predictive markers for response to STZ-based chemotherapy. In the analysis of the RADIANT-1 trial for predictive biomarkers Yao and colleagues suggested that an early decrease of CgA and NSE may serve as a marker of response to everolimus [28]. As the elevation of biomarkers is correlated with tumor load which is an important prognostic marker [29] it appears feasible that a biochemical response predicts OR and correlates with PFS. The observed correlation of a positive SMS-receptor scintigraphy signal indicating a better differentiation with response is intriguing, and it may be argued that SMS-positivity represents a prognostic rather than a predictive marker. However, our study showed a statistically significant association of SMS-positivity with objective response, which may be in line with the observation of O´Toole and co-workers that less well differentiated pNENs with Ki-67 levels >5% lack response to systemic chemotherapy [21].

For advanced, well differentiated pancreatic neuroendocrine tumors chemotherapy represents the therapeutic standard of care. A limited number of cytotoxic drugs have been evaluated in this context. So far, STZ represents the backbone for combination chemotherapy regimens. Former randomized trials revealed superiority of STZ given in combination with Dox or 5-FU as compared to STZ-monotherapy [30, 31]. Due to the cardiotoxicity of Dox the ENETS guidelines favor the administration of STZ in combination with 5-FU [32]. The ORR and DCR and the resulting mPFS and mOS observed in our study are consistent with previous publications. However, there has been considerable controversy regarding the efficacy of STZ-based chemotherapy in pNEN patients. Response rates published so far have ranged between 6% and 69% [5, 7, 8, 30, 33]. The large variability in response rates is thought to be at least partly explained by different standards in assessment of response. As such, in the initial trials the assessment of response was not based on RECIST criteria and in addition to morphological criteria also clinical and biochemical criteria of response were included. This may have resulted in an overestimation of ORR. In our series, a standardized response evaluation by radiological cross-sectional imaging was used.

Our results are comparable with the study of Kouvaraki et al., that reported an ORR of 39% and a mPFS of 18 months using the combination of STZ+Dox+5FU [9]. Furthermore, in the trials mentioned above the different treatment arms were not homogeneous concerning clinicopathological parameters such as age, performance status, grading, tumor load and number of prior treatments factors which are of prognostic relevance and may thus have influenced the outcome to chemotherapy in the different treatment arms. In the study of Kouvaraki et al. a high hepatic tumor load exceeding 75% and the use of chemotherapy as second line treatment was associated with shorter PFS in multivariate analysis. In comparison to this trial, only 20% of our patients received chemotherapy as first-line treatment. Nevertheless, from a clinical point of view chemotherapy remains the first choice in patients in whom response is required e.g. in patients with symptoms due to high tumor mass or in patients who are borderline resectable and may become resectable after induction chemotherapy. Predictive markers as described in our study may help to identify those patients that have the highest benefit from STZ-based chemotherapy. Lack of a biochemical response may thus help in the decision whether to discontinue or proceed with a STZ-based chemotherapy.

In the palliative setting toxicity profile and quality of life in patients undergoing chemotherapy are of utmost importance. In our series, we documented less than 10% grade 3/4 toxicities. In particular, renal failure was one of the limiting toxicities [30, 31], however, occurring in only 3% of the cases, thus confirming the safety of STZ. In addition, the emetogenic and myelosuppressive potential of the combination treatment was less frequent in our patients than reported in prior trials [10, 33, 34] possibly as a result of better standardized supportive management protocols.

With the advent of sunitinib and everolimus, the era of targeted therapies was recently introduced for pancreatic neuroendocrine neoplasms. By improving median PFS from 6 to approximately 12 months, both sunitinib and everolimus were approved in Europe and North America for therapy of metastatic pancreatic NEN [35, 36]. When considering the different therapeutic options, two main questions remain to be elucidated. First, the best choice and sequence of targeted therapies and STZ-based chemotherapy remain to be defined. The ORR of sunitinib and everolimus were only 9% and 5%, respectively, as compared to response rates around 30–40% for STZ-based chemotherapy regimens. At present no comparative trials of chemotherapy versus molecular targeted treatments are available. Trials on the best sequence of treatments, e.g. the SEQTOR trial, a European randomized phase III study investigating STZ+5-FU followed by everolimus versus the reverse sequence, are ongoing and results have to be awaited. Second, so far no means are available for a personalized approach by selecting the most effective therapy for individual patients at the right time by using predictive biomarkers. Up to date mainly prognostic biomarkers such e.g. Ki-67 [24, 37, 38] have been established. However, the role of the proliferation marker Ki-67 as predictor of response to treatment is less well defined. In patients with neuroendocrine carcinoma a Ki-67 value above 55% was associated with better response to platinum based chemotherapy [39] but poorer OS. In patients with well differentiated pNEN it has also been hypothesized that tumors with high Ki-67 may show a better response to chemotherapy [40]. In our study the OR was similar in patients with G1 and G2 tumors although the data on G1 tumors are limited to only 9 G1 cases. Ki-67 values >10% were associated with a shorter mPFS of only 8 months versus 20 months in patients whose tumor had Ki-67 values <10%, however, this was not associated with response to chemotherapy, indicating that in our study the Ki-67 values were prognostic rather than predictive as previously suggested [21].

Finally, our data suggest that objective response (OR) is a favourable prognostic marker for PFS which, however, was not translated into prolonged OS. There are limited data about the relevance of OR as prognostic marker. In fact, our results are in accordance with the PRRT study of Kwekkeboom et al. In this study there were no differences in the survival of patients that revealed either PR or SD [41]. In contrast, PD after PRRT was strongly associated with a poor survival. Patients with a rapid progression after the first reevaluation may thus have a poorer outcome than those with a PD after initial SD or PR, which may partly be explained by a more aggressive biological phenotype as well as by primary or acquired mechanisms of treatment resistance. In this line, Perren and coworkers have shown that loss of DAXX or ATRX is associated with chromosomal instability and shorter survival times in patients with pNENs [42]. In the same way loss of MGMT protein was associated with an adverse outcome in pNEN patients, this prognostic value, however, was not independent from grade and stage in multivariate analysis [43]. These results suggest, that among well differentiated pNENs subtypes exist that show a more aggressive and unfavourable course, such those that are DAXX- and ATRX-negative or show a loss of MGMT-protein expression.

In conclusion, our data support the use of STZ-based chemotherapy in patients with advanced pancreatic NENs. Prognostic and predictive subtypes of pNENs exist and our data based on standard clinicopathological characteristica suggest that positivity of SMS-receptor scintigraphy as a surrogate parameter of better differentiation and biochemical response may be useful to predict response to therapy and thus contribute to personalize treatment. However, based on the retrospective nature and the limited number of included patients in this study, our findings need to be validated in well-designed prospective clinical trials.

## Supporting Information

S1 Table Patient data for statistical analyses. (XLS)
